# From cyberbullying victimization to perpetration: the mediating role of attitudes among middle-aged adults

**DOI:** 10.3389/fpsyg.2026.1753887

**Published:** 2026-02-03

**Authors:** Myeong-Sook Yoon, HyunKyoung Yu, Heesoo Kim

**Affiliations:** Department of Social Welfare, Jeonbuk National University, Jeonju, Republic of Korea

**Keywords:** attitudes toward cyberbullying, cyberbullying perpetration, cyberbullying victimization, middle-aged adults, mediating role

## Abstract

With the widespread use of digital technologies, cyberbullying has emerged as a significant social problem across all age groups, including middle-aged adults. While most prior research has focused on adolescents and young adults, this study investigates the relationship between cyberbullying victimization and perpetration among middle-aged individuals (ages 40–64), and examines the mediating role of attitudes toward cyberbullying. Using nationally representative data from the 2022 Cyberbullying Survey conducted by the Korea Information Society Agency, responses from 4,105 participants were analyzed. The findings revealed that experiences of cyberbullying victimization were significantly associated with a higher likelihood of perpetration, and this relationship was partially mediated by the respondent’s attitude toward cyberbullying. Specifically, those with more frequent victimization experiences tended to adopt more permissive attitudes toward cyberbullying, which in turn increased the probability of engaging in perpetration. These results highlight the cyclical nature of cyberbullying and underscore the importance of attitude transformation as a preventive intervention strategy, particularly for digital-vulnerable middle-aged populations.

## 1 Introduction

Digital technology has facilitated widespread online communication among individuals of all age groups. While the majority of extant research on cyberbullying has focused on adolescents and young adults, recent studies have begun to explore its occurrence among middle-aged adults as well (Kowalski et al., 2019; Jenaro et al., 2018). Surveys conducted in various countries have revealed that cyberbullying is not confined to any specific age demographic and increasingly manifests during adulthood. This issue has emerged as a topic of growing social concern among middle-aged adults, who represent a demographic with high rates of internet and smart device use (Pew Research Center, 2020; Eurostat, 2020). Park and Bae's (2025) study revealed that a significant proportion of adults over 40 have been subjected to cyberbullying, either as victims or perpetrators. According to Korea Communications Commission and National Information Society Agency (2024), internet usage among middle-aged adults has exceeded 90%. These findings suggest that middle-aged adults constitute an active digital population increasingly exposed to diverse forms of cyber violence. This increase in internet access has, in turn, led to a diversification of cyberviolence, including online conflicts, defamation, and malicious comments.

A growing body of discourse has emerged across various countries regarding cyberbullying, with particular emphasis on the experiences of middle-aged adults. According to findings from the Pew Research Center (2020), the rate of cyberbullying against middle-aged adults is lower than that against young adults. However, the incidence of cybercrimes targeting this group—such as phishing and fraud—is on the rise. A 2020 survey by Eurostat reported that, in 2019, middle-aged adults accounted for 33% of internet security incident victims in Europe, with especially high rates observed in Denmark (50%) and France (46%). Together, these findings indicate that cyber-related risks affecting middle-aged adults are both quantitatively significant and qualitatively distinct from those experienced by younger populations.

Middle-aged adults often occupy key roles in families, workplaces, and communities (Kowalski et al., 2019). However, this demographic may lack sufficient understanding and proficiency in digital media, making them more vulnerable to online interactions than face-to-face communication (Butkovic et al., 2020). A policy report by the Seoul 50 + Foundation (2023) found that exposure to cyberbullying in this group is linked to adverse psychological responses such as emotional withdrawal, a desire for revenge, and social isolation. The report highlights the need for preventive education and counseling services to address these issues. In particular, in anonymous online environments—such as social media, messaging platforms, and online communities—middle-aged adults are increasingly becoming targets of attacks or conflict, often without recognizing it. This type of harm is gradually emerging as a widespread social problem among middle-aged adults, who often face cyber-related conflicts without adequate digital awareness or institutional support.

Research on cyberbullying targeting middle-aged adults remains in its early stages. Most existing studies on adult cyberbullying have focused on young adults or college students, with limited research specifically addressing middle-aged populations (Turan et al., 2011; Kokkinos et al., 2014; Doane et al., 2014; Cénat et al., 2019). In their review of the literature, Jenaro et al. (2018) similarly noted that the majority of studies on adult cyberbullying center on college students. This highlights the lack of research on diverse adult age groups, particularly middle-aged individuals. Barlett and Chamberlin (2017) conducted a study on cyberbullying among individuals aged 11 to 75, revealing that cyberbullying patterns vary significantly across age groups. These findings underscore the need for research that examines how older adults adapt to the digital environment and how this adaptation influences their relationships, mental health, and emotional well-being. Despite these age-related differences, empirical research specifically examining the mechanisms of cyberbullying among middle-aged adults remains scarce.

The consequences of cyberbullying extend beyond interpersonal conflict, encompassing serious psychological outcomes such as diminished self-esteem, depression, and suicidal ideation (Cénat et al., 2019; Alsawalqa, 2021). Middle-aged adults play pivotal roles within their families, workplaces, and communities, and the mental health challenges they face as a result of cyberbullying can have broad societal implications (Schodt et al., 2021). The psychological effects of cyberbullying may persist over time and across contexts, often leading to prolonged distress and social isolation (Hinduja and Patchin, 2010; Petri, 2022; Neuhaeusler, 2024). These effects are particularly concerning given the elevated risk of suicide observed among both victims and perpetrators of cyberbullying (Fekih-Romdhane et al., 2024). As such, addressing cyberbullying requires a multifaceted approach that incorporates both preventive and therapeutic interventions.

Cyberbullying is influenced by a range of factors, including emotional distress, lack of empathy, aggression, moral disengagement, insufficient social support, and problematic social media use (Kırcaburun et al., 2019; Doane et al., 2014; Kokkinos et al., 2014; Schodt et al., 2021). Individuals who lack empathy are more likely to justify or tolerate cyberbullying, thereby increasing the likelihood of engaging in such behavior. Similarly, those who experience loneliness or possess a limited social network are more prone to display online aggression (Wu, 2022).

Research has demonstrated a strong correlation between experiences of cyberbullying victimization and subsequent perpetration (Hinduja and Patchin, 2010; Ramos Salazar, 2021; Fekih-Romdhane et al., 2024). Feelings of anger and frustration resulting from victimization can foster a desire for retaliation, escalating aggressive online behavior (Balakrishnan, 2015). Of particular interest is the “bully-victims” group, in which cyberbullying victimization and perpetration coexist. This group tends to exhibit emotional desensitization, reducing sensitivity to cyberbullying and increasing susceptibility to engaging in such behavior (Ramos Salazar, 2021; Ma et al., 2024). Additionally, experiences of powerlessness, social alienation, or disconnection may further contribute to the transition from victim to perpetrator (Reiter-Scheidl et al., 2018). These findings suggest the need to identify psychological mechanisms that explain how victimization experiences translate into perpetration behaviors in adulthood.

The cyclical transition from victimization to perpetration can be explained through General Strain Theory and Social Learning Theory. According to General Strain Theory, individuals may engage in deviant behavior as a coping mechanism in response to accumulated stress and negative emotional experiences (Kim, 2021). While Social Learning Theory has traditionally emphasized the role of observation and imitation in explaining aggressive behavior among children and adolescents, recent research suggests that learning processes in adulthood operate primarily through cognitive internalization rather than direct behavioral imitation. Among adults, particularly middle-aged individuals, exposure to cyber aggression may shape internal norms, attitudes, and moral evaluations that subsequently guide behavior. In this sense, Social Learning Theory can be extended to explain how attitudes toward cyberbullying—rather than overt imitation—serve as a mechanism through which victimization experiences increase the likelihood of perpetration (Chen and Chen, 2025). These frameworks help explain how, particularly among middle-aged adults, the boundaries between victim and perpetrator may blur, leading to reciprocal or transitional patterns of cyberbullying (Lucas, 2018).

Attitudes toward cyberbullying have been shown to significantly influence the pathway through which victimization experiences lead to perpetration (Doane et al., 2014; Ma et al., 2024). In adulthood, attitudes toward cyberbullying reflect accumulated social norms, moral reasoning, and prior digital experiences, making them particularly salient mechanisms for explaining behavioral transitions among middle-aged adults. Meter and Bauman (2018) proposed that individuals’ attitudes play a pivotal role in the process of rationalizing behaviors that contravene moral standards through a phenomenon known as moral disengagement. Such attitudes emerge as key psychological variables that explain the level of justification for deviant or aggressive behavior. In the context of online environments, this correlation between attitudes and behaviors becomes particularly pronounced. The digital environment is characterized by anonymity and non-face-to-face interaction, which serve to lower barriers that prevent internal attitudes from manifesting as external behavior. This, in turn, increases the likelihood of alignment between attitudes and actual behavior (Doane et al., 2014). Barlett et al. (2017) developed a theoretical model, the Barlett-Gentile Cyberbullying Model (BGCM), to predict cyberbullying perpetration. Their research found that adults’ attitudes toward cyberbullying act as a significant mediating variable in cyberbullying perpetration. Consequently, individuals who exhibit tolerant or justifying attitudes toward cyberbullying are more likely to engage in perpetration. Park et al. (2021) conducted an extensive analysis of studies on cyberbullying centered on China, Japan, South Korea, Taiwan, and Hong Kong. Utilizing a rigorous empirical approach, the researchers effectively demonstrated the influence of attitudes that trivialize or justify cyberbullying on subsequent perpetration behaviors. A similar finding was reported by Park and Bae (2025) in a study targeting adult populations.

The attitudes towards cyberbullying vary depending on variables such as gender, age, victimization experience, and morality. These differences in attitudes influence the motives and intensity of actual cyberbullying behavior (Barlett and Chamberlin, 2017). In South Korea, a study on cyberbullying targeting adults aged 20–50 (Kim, 2021) found that older age was associated with less tolerant attitudes toward cyberbullying and lower involvement in perpetrating such behavior. This suggests that middle-aged and older adults may form more negative attitudes toward cyberbullying based on relatively conservative social values and mature moral judgment. However, as Jenaro et al. (2018) have noted, adults’ attitudes toward cyberbullying may be characterized by inconsistency, owing to deficiencies in digital literacy, conceptual confusion, and a paucity of contextual understanding of cyberbullying. This inconsistency suggests that attitudes toward cyberbullying among middle-aged adults may be particularly susceptible to situational factors and prior victimization experiences.

In essence, attitudes toward cyber violence function as a pivotal predictor of perpetration behavior and serve as a crucial cognitive mediator in understanding the relationship between middle-aged adults’ experiences of cyber violence victimization and perpetration (Kim, 2021). Middle-aged adults represent a demographic that has internalized social responsibility and norms. However, they also constitute a transitional generation that is experiencing sociocultural turmoil due to the digital transformation. The interplay between these two factors contributes to the multifaceted formation of attitudes toward cyber violence. Despite the theoretical importance of attitudes, empirical studies explicitly testing their mediating role among middle-aged adults remain limited. However, there is a paucity of research that has empirically validated the mediating role of attitudes in the relationship between experiences of cyber violence victimization and perpetration behaviors among middle-aged adults. Therefore, the objective of this study is to analyze the influence of experiences with cyber violence victimization on perpetration behaviors among middle-aged adults (aged 40–64) and empirically verify the mediating effect of attitudes toward cyber violence in this relationship. This research will provide a solid foundation for structurally understanding cyber violence issues among middle-aged adults and developing generation-specific prevention and intervention strategies in the future.

## 2 Methods

This study investigates whether attitudes toward cyberbullying mediate the relationship between cyberbullying victimization and perpetration among middle-aged adults.

### 2.1 Research model

**Figure 1:**
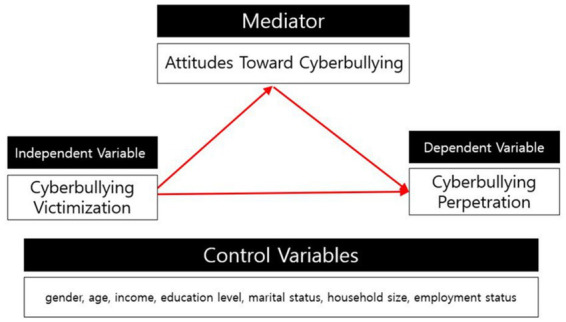
The research model.

This study investigates whether attitudes toward cyberbullying mediate the relationship between cyberbullying victimization and perpetration among middle-aged adults. The research model is presented in Figure 1.

### 2.2 Research subjects and data collection methods

**Figure 2:**
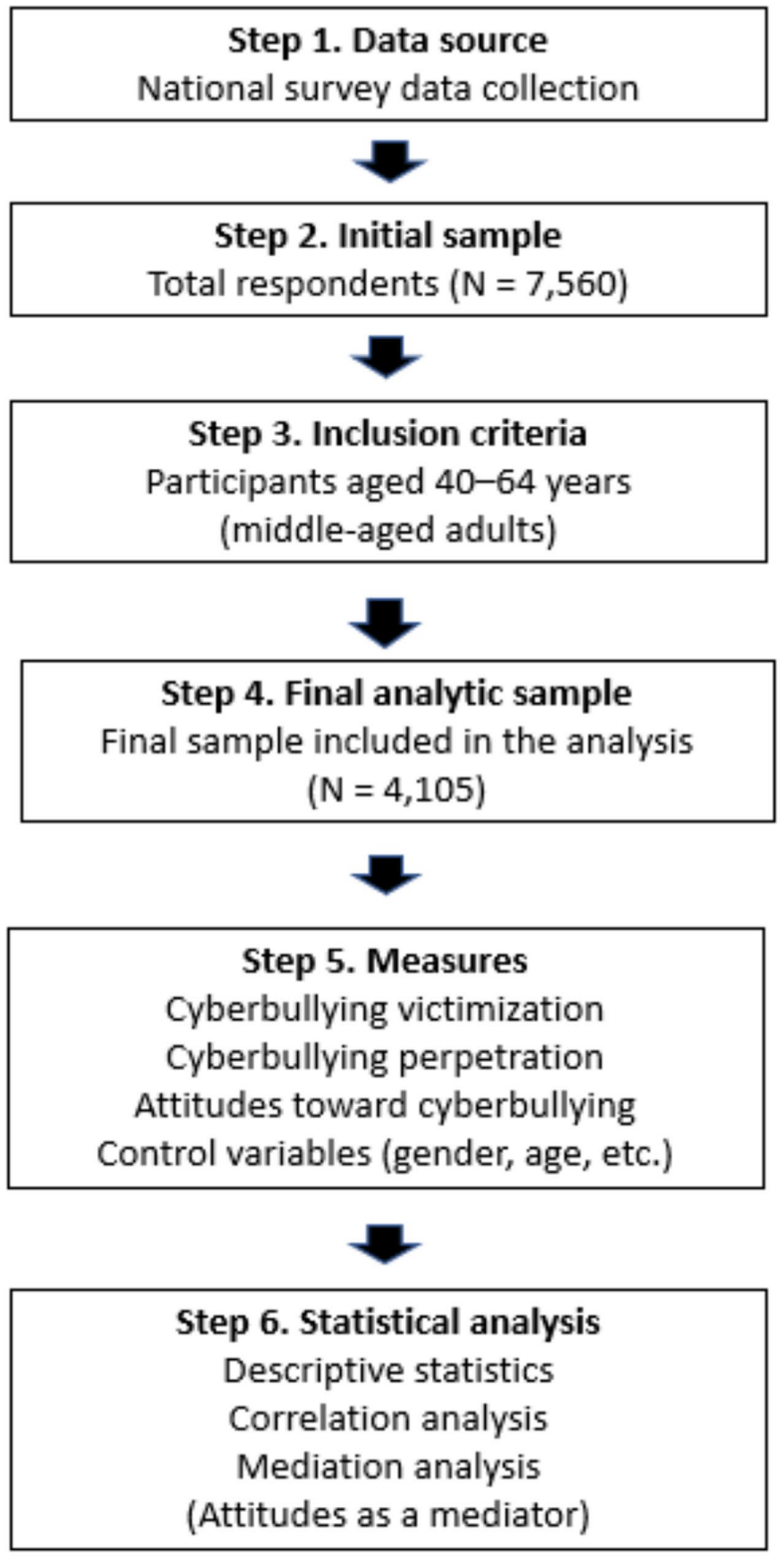
Flowchart of the study design and participant selection process.

Figure 2 illustrates the participant selection process and overall study procedure.

This study utilized adult data from the 2022 Cyberbullying Survey conducted by the Korea Intelligent Information Society Promotion Agency. In this study, middle-aged adults were defined as individuals aged 40–64 years. This age range was determined based on classifications commonly used in national statistics and prior research in Korea, where adulthood is often divided into young adulthood, middle adulthood, and older adulthood. The upper limit of 64 years was set to distinguish middle-aged adults from older adults, who may exhibit different patterns of digital use and psychosocial characteristics.

The survey, originally launched in 2013 to inform cyberbullying prevention and response policies, was officially designated as a national statistical survey in 2021. For the purpose of this study, 4,105 individuals aged 40 to 64 were selected from the total sample of 7,560 respondents. To ensure compliance with research ethics, this study received approval from the Institutional Review Board (IRB No. JBNU 2024–09-011).

This survey was conducted using a standardized questionnaire and data collection procedure administered by the Korea Intelligent Information Society Promotion Agency. The sampling process followed a stratified sampling design based on key demographic characteristics, including age, gender, and region, to ensure national representativeness. Data collection was carried out through a systematic and standardized process, and survey weights were applied in the original dataset to reflect the population distribution. As a result, the data are considered to be sufficiently representative of the adult population in Korea.

Although the proportion of participants aged 50–64 was relatively higher than that of younger age groups within the middle-aged sample, age was included as a control variable in all regression analyses. This approach allowed us to account for potential age-related effects within the middle-aged group and to minimize the influence of age distribution on the study results.

### 2.3 Measurement tools

#### 2.3.1 Dependent variable: cyberbullying perpetration

Cyberbullying perpetration was assessed using a structured survey that measured participants’ engagement in eight subtypes of cyberbullying: cyber verbal abuse, defamation, stalking, sexual abuse, disclosure of personal information, exclusion, extortion, and coercion. This measurement was adapted and refined by the Korea Intelligent Information Society Promotion Agency (2018), based on scales developed by Meter and Bauman (2018) and Barlett and Gentile (2012).

Participants rated the frequency of each behavior over the past year on a 4-point scale ranging from “none at all” (9 points) to “almost daily” (1 point), which was then reverse-coded to a 1–4 scale. Total scores ranged from 8 to 40, with higher scores indicating more frequent perpetration. The reliability of the scale was high (Cronbach’s *α* = 0.8551).

#### 2.3.2 Independent variable: cyberbullying victimization

Cyberbullying victimization was assessed by measuring participants’ experiences with eight subtypes: cyber verbal abuse, defamation, stalking, sexual violence, disclosure of personal information, bullying, extortion, and coercion. The scale was adapted from Meter and Bauman (2018) and modified by the Korea Intelligent Information Society Promotion Agency (2018).

Participants rated the frequency of each experience over the past year on a 4-point scale ranging from “none at all” (9 points) to “almost every day” (1 point), which was then reverse-coded to a 1–4 scale. Total scores ranged from 8 to 40, with higher scores indicating more frequent victimization. The internal consistency of the scale was acceptable (Cronbach’s *α* = 0.7572).

#### 2.3.3 Mediating variable: attitudes toward cyberbullying

Attitudes toward cyberbullying were assessed based on the extent to which respondents perceived specific cyberbullying behaviors as problematic. Participants evaluated eight subtypes—cyber verbal abuse, defamation, stalking, sexual violence, disclosure of personal information, exclusion, extortion, and coercion—using a 4-point scale ranging from “not at all problematic” (1 point) to “very problematic” (4 points).

The scale was adapted from Meter and Bauman (2018) and further modified by the Korea Intelligent Information Society Promotion Agency (2018). Total scores ranged from 8 to 32, with lower scores reflecting more tolerant attitudes and higher scores indicating stronger disapproval of cyberbullying. The scale demonstrated high internal consistency (Cronbach’s *α* = 0.8670).

#### 2.3.4 Control variables: gender, age, income, education level, marital status, number of household members, and employment status

Control variables were selected based on prior research and include gender (Marcum et al., 2014), age (Barlett and Chamberlin, 2017; Kowalski et al., 2019), income (Duggan, 2017), education level (Duggan, 2017), marital status (López-Castro and Priegue, 2019), number of household members (Chen et al., 2017), and employment status (Duggan, 2017).

### 2.4 Analysis method

All analyses were conducted using Stata/MP 17.0. First, frequency and descriptive statistics were used to examine the general characteristics of the participants. Second, Pearson correlation analysis was performed to assess the relationships among key variables and to check for multicollinearity. Third, regression analysis was conducted to test the mediating effect of attitudes toward cyberbullying on the relationship between victimization and perpetration. The Sobel test was additionally used to verify the significance of the mediating effect.

## 3 Results

### 3.1 Sociodemographic characteristics of participants

Among the 4,105 participants, 1,927 (46.94%) were male and 2,178 (53.06%) were female, indicating a slightly higher proportion of females. In terms of age, 1,538 individuals (37.47%) were aged 40–49, and 2,567 (62.53%) were aged 50–64, showing a larger proportion of older adults in the sample.

Regarding monthly income, 86 participants (2.10%) earned less than 1 million won, 161 (3.92%) earned 1–2 million won, 554 (13.50%) earned 2–3 million won, and 1,097 (26.72%) earned 3–4 million won, the largest income group. Additionally, 842 (20.51%) earned 4–5 million won, 795 (19.37%) earned 5–6 million won, 299 (7.28%) earned 6–7 million won, 143 (3.48%) earned 7–8 million won, and 128 (3.12%) earned 8 million won or more.

In terms of education, 204 participants (4.97%) had a middle school education or less, 2,060 (50.18%) had a high school diploma or less, and 1,841 (44.85%) held a college degree or higher. With respect to marital status, 3,420 (83.31%) were married, and 685 (16.69%) were unmarried. The most common household size was two people (1,331; 32.42%), followed by three (1,007; 24.53%), four (933; 22.73%), one (657; 16.00%), and five or more (177; 4.31%). Regarding employment status, 3,232 participants (78.73%) were employed, while 873 (21.27%) were unemployed (Table 1).

**Table 1:** General characteristics of the participants.

Variable	Category	Frequency (N)	Percentage (%)
Gender	Male	1,927	46.94
	Female	2,178	53.06
Age	40–49	1,538	37.47
	50–64	2,567	62.53
Income (monthly)	Less than $2,143	801	19.52
	$4,286–$5,000	2,734	66.6
	$4,286 or more	570	13.88
Education	Middle school or less	204	4.97
	High school graduate or less	2,060	50.18
	College or higher	1,841	44.85
Marital status	Unmarried	685	16.69
	Married	3,420	83.31
Household size	1 person	657	16.00
	2 persons	1,331	32.42
	3 persons	1,007	24.53
	4 persons	933	22.73
	5 persons or more	177	4.31
Employment status	Employed	3,232	78.73
	Unemployed	873	21.27
Total		4,105	100

### 3.2 Correlation between key variables

Pearson correlation analysis was conducted to examine the relationships among key variables and to assess multicollinearity. All correlation coefficients were below 0.80, and variance inflation factor (VIF) values were below 10 (maximum VIF = 1.25), indicating no multicollinearity concerns (see Table 2).

**Table 2:** Correlations and descriptive statistics among variables (N = 4,105).

	1. Cyberbullying perpetration	2. Cyberbullying victimization	3. Attitudes toward cyberbullying
1. Cyberbullying Perpetration	1		
2. Cyberbullying Victimization	0.5644***	1	
3. Attitudes Toward Cyberbullying	−0.2196***	−0.1511***	1
Mean	8.078	8.147	29.498
Standard Deviation	0.624	0.848	2.706

### 3.3 Mediating effect of attitudes toward cyberbullying in the relationship between victimization and perpetration

Table 3 presents the results for Step 1 and Step 2, and Table 4 presents the results for Step 3 of the mediation analysis examining whether attitudes toward cyberbullying mediate the relationship between cyberbullying victimization and perpetration among middle-aged adults, after controlling for demographic variables.

**Table 3:** Regression results for step 1 and step 2 of the mediation analysis examining the relationship between cyberbullying victimization, attitudes toward cyberbullying, and perpetration among middle-aged adults (*n* = 4,105).

Variables		Step 1 (Victim → Attitude)			Step 2 (Victim → Perpetration)		
		B(SE)	*t*	*β*	*B* (SE)	*t*	*β*
Independent Variable	Cyberbullying victimization	−0.476 (0.049)	−9.660***	−0.149	0.415 (0.010)	43.700***	0.564
Mediating Variable	Attitudes toward cyberbullying						
Control variables	Gender (ref: male)	0.250 (0.091)	2.760**	0.046	−0.010 (0.017)	−0.590	−0.008
	Age	−0.062 (0.095)	−0.650	−0.011	−0.025 (0.018)	−1.380	−0.020
	Income	−0.054 (0.030)	−1.790	−0.033	−0.002 (0.006)	−0.370	−0.006
	Education level	−0.092 (0.082)	−1.120	−0.020	−0.010 (0.016)	−0.640	−0.009
	Marital status (ref: married)	0.030 (0.134)	0.230	0.004	0.022 (0.026)	0.870	0.013
	Household size	−0.041 (0.044)	−0.920	−0.017	0.006 (0.009)	0.720	0.011
	Employment status (ref: employed)	−0.039 (0.112)	−0.350	−0.006	−0.010 (0.022)	−0.470	−0.007
Constant		33.87 (0.475)	71.37***	.	4.72 (0.092)	51.59***	.
*R* ^2^		2.79			31.94		
adj. *R*^2^		2.60			31.80		
*F*		14.68***			240.23***		

B = Coeffcients, β = Standardized Coeffcients, SE = Standard Error. *p < 0.05, **p < 0.01, and ***p < 0.001.

**Table 4:** Regression results for Step 3 of the mediation analysis examining the mediating effect of attitudes toward cyberbullying on the relationship between victimization and perpetration among middle-aged adults (*n* = 4,105).

Variables		Step 3 (Victim + Attitude → Perpetration)		
		B(SE)	*t*	*β*
Independent variable	Cyberbullying victimization	0.400 (0.009)	42.210***	0.544
Mediating variable	Attitudes toward cyberbullying	−0.032 (0.003)	−10.700***	−0.138
Control variables	Gender (ref: male)	−0.002 (0.017)	−0.130	−0.002
	Age	−0.027 (0.018)	−1.510	−0.021
	Income	−0.004 (0.006)	−0.670	−0.010
	Education level	−0.013 (0.016)	−0.830	−0.012
	Marital status (ref: married)	0.023 (0.025)	0.910	0.014
	Household size	0.005 (0.008)	0.570	0.009
	Employment status (ref: employed)	−0.011 (0.021)	−0.530	−0.007
Constant		5.80 (0.135)	42.89***	.
*R* ^2^		33.79		
adj. *R*^2^		33.64		
*F*		232.17***		

B = Coeffcients, β = Standardized Coeffcients SE = Standard Error. *p < 0.05, **p < 0.01, ***p < 0.001.

In Step 1, cyberbullying victimization significantly predicted attitudes toward cyberbullying (*β* = −0.149, *p* < 0.001), explaining 2.6% of the variance (*F* = 14.68, *p* < 0.001). In Step 2, victimization also significantly predicted perpetration (*β* = 0.564, *p* < 0.001), with an explanatory power of 31.8% (*F* = 240.23, *p* < 0.001). In Step 3, both victimization and attitudes were entered simultaneously. Victimization (*β* = 0.544, *p* < 0.001) and attitudes (*β* = 0.138, *p* < 0.001) each had significant effects on perpetration, explaining 33.64% of the variance (*F* = 232.17, *p* < 0.001). These results confirm that attitudes toward cyberbullying partially mediate the relationship between victimization and perpetration among middle-aged adults.

A Sobel test was conducted to verify the significance of the mediating effect of attitudes toward cyberbullying. As shown in Table 5, the *Z*-value was 7.182 (*p* < 0.001), exceeding the critical value of 1.96. This result confirms that the mediating effect of attitudes is statistically significant in the relationship between cyberbullying victimization and perpetration.

**Table 5:** Sobel test results.

Path	Sobel test statistic	*p*
Cyberbullying victimization → Attitudes toward cyberbullying → Perpetration	7.182	<0.001

## 4 Discussion

This study examined the relationship between cyberbullying victimization and perpetration among middle-aged adults and tested whether attitudes toward cyberbullying mediated this relationship. The findings yield three key implications.

First, victimization experiences were significantly associated with individuals’ attitudes toward cyberbullying. Specifically, increased exposure to cyberbullying was associated with more permissive attitudes, reflecting a lower level of disapproval toward cyberbullying behaviors. This suggests that victimization may be related to shifts in evaluative attitudes toward cyberbullying, rather than indicating changes in unmeasured cognitive or emotional processes. These results are consistent with previous findings that repeated victimization can foster permissive attitudes (Eraslan-Çapan and Bakioğlu, 2020), but contrast with studies indicating that victimization may instead heighten critical awareness and negative attitudes (Chen et al., 2024).

Second, cyberbullying victimization was positively associated with perpetration. In other words, individuals reporting higher levels of victimization were more likely to report engaging in perpetration. This pattern suggests a continuity or overlap between victimization and perpetration roles in cyber contexts, rather than a strictly unidirectional process. The finding is consistent with prior research documenting the close association between experiences of being victimized and subsequent involvement in cyberbullying behaviors (Hinduja and Patchin, 2010; Ramos Salazar, 2021; Fekih-Romdhane et al., 2024; Ma et al., 2024), and extends this evidence to middle-aged adults, a population that has received comparatively limited attention in the cyberbullying literature.

Third, attitudes toward cyberbullying were found to partially mediate the relationship between victimization and perpetration. Greater victimization was associated with more permissive attitudes toward cyberbullying, which were in turn associated with a higher likelihood of reported perpetration. Recent adult-focused research provides additional evidence for the role of attitudes in cyberbullying processes. Chen and Chen (2025) demonstrated that attitudes toward online aggression serve as a key psychological pathway linking adverse online experiences to aggressive responses among adults. This result is consistent with theoretical perspectives from social psychology, including the theory of planned behavior (Ajzen, 1991), and aligns with empirical findings highlighting the role of attitudinal processes underlying cyberbullying (Doane et al., 2014; Barlett et al., 2017; Park et al., 2021). These findings underscore the importance of addressing not only behavioral outcomes but also the attitudinal processes identified in this study when designing intervention strategies for middle-aged adults involved in cyberbullying.

Given the increasing digital engagement of middle-aged adults, intervention strategies informed by the present findings should include digital ethics education and attitude-focused programs. Rather than making assumptions about levels of awareness or preparedness, the results of this study suggest that preventive, education-based approaches that address attitudinal processes associated with cyberbullying may be particularly important. Such approaches may complement victim-focused responses by targeting the mechanisms through which victimization is linked to perpetration. The development of tailored interventions and policy support grounded in empirically identified pathways is therefore necessary to address the needs of this population.

By empirically demonstrating the mediating role of attitudes, this study highlights that cyberbullying victimization is not merely a passive experience, but is associated with processes through which individuals may come to justify and engage in aggressive behavior. These findings call for a multifaceted approach to reduce cyberbullying perpetration among middle-aged adults, one that addresses both experiences of victimization and the attitudinal processes identified in this study that may contribute to the continuation of aggressive behavior.

A key implication of this study is the importance of early recognition and intervention in cyberbullying victimization. Given that victimization was significantly associated with perpetration, timely responses to initial incidents may play an important role in reducing the risk of escalation. Prior research suggests that adult victims often attempt to collect evidence but may hesitate to access formal support systems such as legal or psychological services (Kaluarachchi et al., 2021). Rather than attributing this pattern to unmeasured levels of awareness, it may reflect structural or practical barriers to accessing available response mechanisms among middle-aged adults. Accordingly, policies that strengthen early detection and facilitate access to intervention and support services should be prioritized.

Specifically, the establishment of cyberbullying counseling and psychological support programs that are coordinated with existing community infrastructure—such as local welfare centers, senior centers, and resident governance hubs—may help improve access to appropriate support services. Additionally, the use of digital reporting systems within online platforms may reduce barriers to help-seeking by facilitating initial contact with support resources. When necessary, such systems may be linked to legal, psychological, and emotional support services, enabling a more comprehensive and coordinated response.

International examples provide illustrative models of integrated intervention approaches. For example, the UK- and US-based Cybersmile Foundation has developed 24/7 online assistance tailored to victims’ needs through an AI-based platform, offering access to legal, emotional, and social support (Cybersmile Foundation, 2024). Similarly, Australia’s “Adult Resilience for Life” program incorporates cognitive-behavioral strategies to support adults in managing emotions such as anxiety and anger (Parks Community Network, 2025). In the United States, StopBullying.gov provides step-by-step guidance for adults on evidence preservation, platform-level reporting, and legal engagement (U.S. Department of Health and Human Services, 2025). Taken together, these examples suggest the potential value of multi-tiered intervention frameworks that combine early response, emotional regulation, and access to legal recourse.

Second, education aimed at modifying attitudes toward cyberbullying should be strengthened. This study demonstrated that experiences of cyberbullying victimization were associated with more permissive attitudes, which were in turn associated with higher levels of perpetration. This suggests that attitude change represents an important target for prevention efforts. Rather than making assumptions about familiarity with digital interactions, the present findings indicate that awareness-building and targeted educational initiatives focused on attitudinal processes may be beneficial for middle-aged adults.

Educational content may usefully address the definition, types, legal implications, and real-world cases of cyberbullying, and be delivered through settings such as regional lifelong learning centers, workplace training, and user-friendly online platforms. In particular, the present findings suggest the value of customized content and accessible interface design tailored to the digital environments frequented by middle-aged users—such as portal communities, messenger apps, and social media—are needed. In the Korean context, recent evidence highlights the importance of attitudinal factors in adult cyberbullying. Park and Bae (2025) reported that internet ethics and attitudes toward online behavior significantly shape the association between cyberbullying victimization and perpetration among Korean adults. These attitudinal patterns may be further shaped by sociocultural characteristics of Korean society, including collectivist social norms, strong family and workplace role expectations, and relatively conservative moral values, which can influence how cyberbullying behaviors are evaluated and justified. In South Korea, the Korea Communications Commission and the Korea Intelligent Information Society Agency have developed adult-targeted programs such as the “Healthy Cyber Ethics Culture Creation Project,” which promote responsible internet use (Korea Communications Commission and National Information Society Agency, 2023). Similarly, the Korea Gender Equality Education Promotion Institute provides educational content on digital sexual violence prevention (Korea Gender Equality Education Promotion Institute, 2025).

However, many domestic initiatives have primarily emphasized preventive awareness, with relatively less focus on structured approaches to attitude change, legal literacy, or emotional coping. In contrast, several international programs illustrate examples of more practice-oriented educational approaches. New Zealand’s Netsafe provides adult-targeted workshops and resources focused on recognizing and responding to cyberbullying (Netsafe, 2025). The U.S.-based NetCE delivers specialized training to professionals on identifying victims, understanding legal procedures, and implementing institutional responses (NetCE, 2025). Taken together, these examples suggest the potential value of moving beyond purely informational delivery toward educational approaches that support attitudinal and behavioral change among middle-aged adults.

Finally, the findings further indicate that cyberbullying victimization among middle-aged adults is associated with higher levels of perpetration through attitudinal processes. This suggests that victimization is not merely a passive experience, but may be related to changes in how cyberbullying behaviors are evaluated, which can be linked to subsequent behavioral responses. Accordingly, psychosocial interventions that address attitude adjustment and emotion regulation may represent a valuable approach for this population.

Group counseling and digital interventions—such as cognitive-behavioral communication training, anger management, and emotion recognition programs—may be considered as potential intervention options. Previous research has provided evidence supporting the efficacy of online cognitive behavioral therapy (CBT) in managing problematic emotional responses. For example, Howie and Malouff (2014) showed that online CBT significantly reduced problematic anger in adults. Similarly, Pop et al. (2025) reported that an online emotion regulation program significantly improved emotional awareness and control, reducing both state and trait anger.

Based on these findings, online CBT programs tailored to middle-aged adults who have experienced cyberbullying may be a promising intervention approach. Such programs could be complemented by group-based interventions and simulation-based education aimed at supporting emotional regulation and empathy.

Beyond intervention approaches, individual characteristics may also play a role in shaping cyber violence behaviors. Educational attainment may also play an important role in shaping attitudes and behaviors related to cyber violence. Although education level was included as a control variable in the present study, future research could further examine whether education level has a moderating or mediating effect on the relationship between cyber violence victimization and perpetration. Such analyses may provide a more nuanced understanding of how educational background influences cyber violence among middle-aged adults.

## 5 Conclusion and implications

This study extends existing research on cyberbullying by focusing on middle-aged adults—an underexplored demographic in the literature. Through empirical analysis, it identifies the psychological mechanisms underlying cyberbullying in this population and highlights the mediating role of attitudes in the transition from victimization to perpetration. In doing so, the study offers meaningful theoretical insight into the cyclical nature of cyberbullying beyond adolescence.

By situating middle-aged adults as a digitally vulnerable group, the study also emphasizes the risks posed by increased digital engagement in the absence of sufficient structural and psychosocial support mechanisms. These findings offer practical implications for the development of targeted intervention strategies, including attitude-focused education, early detection systems, and psychosocial support tailored to the needs of this demographic.

## 6 Limitations

This study has several limitations. First, as it is based on cross-sectional data, causal inferences are limited. Longitudinal studies are needed to capture changes over time and to more accurately establish causal relationships. Second, experiences and attitudes related to cyberbullying were assessed through self-report measures, which may be subject to biases such as social desirability and memory distortion. This could result in the underreporting or overreporting of actual experiences. Third, although the study included key variables in its analysis, it may not have fully accounted for other personal or contextual factors that influence cyberbullying behavior. Future research should incorporate additional control variables and consider more comprehensive modeling approaches to better understand the mechanisms underlying cyberbullying among middle-aged adults.

## Data Availability

The raw data supporting the conclusions of this article will be made available by the authors, without undue reservation.
